# Applications of Variability Analysis Techniques for Continuous Glucose Monitoring Derived Time Series in Diabetic Patients

**DOI:** 10.3389/fphys.2018.01257

**Published:** 2018-09-06

**Authors:** Klaus-Dieter Kohnert, Peter Heinke, Lutz Vogt, Petra Augstein, Eckhard Salzsieder

**Affiliations:** ^1^Institute of Diabetes “Gerhardt Katsch”, Karlsburg, Germany; ^2^Diabetes Service Center, Karlsburg, Germany; ^3^Heart and Diabetes Medical Center, Karlsburg, Germany

**Keywords:** variability analysis techniques, continuous glucose monitoring, glucose time series, indices of non-linear and fractal dynamics, multiscale entropy, Poincaré plots, detrended fluctuation analysis, glycemic control of diabetes

## Abstract

Methods from non-linear dynamics have enhanced understanding of functional dysregulation in various diseases but received less attention in diabetes. This retrospective cross-sectional study evaluates and compares relationships between indices of non-linear dynamics and traditional glycemic variability, and their potential application in diabetes control. Continuous glucose monitoring provided data for 177 subjects with type 1 (*n* = 22), type 2 diabetes (*n* = 143), and 12 non-diabetic subjects. Each time series comprised 576 glucose values. We calculated Poincaré plot measures (SD1, SD2), shape (SFE) and area of the fitting ellipse (AFE), multiscale entropy (MSE) index, and detrended fluctuation exponents (*α1, α2*). The glycemic variability metrics were the coefficient of variation (%CV) and standard deviation. Time of glucose readings in the target range (TIR) defined the quality of glycemic control. The Poincaré plot indices and *α* exponents were higher (*p* < 0.05) in type 1 than in the type 2 diabetes; SD1 (mmol/l): 1.64 ± 0.39 vs. 0.94 ± 0.35, SD2 (mmol/l): 4.06 ± 0.99 vs. 2.12 ± 1.04, AFE (mmol^2^/l^2^): 21.71 ± 9.82 vs. 7.25 ± 5.92, and α1: 1.94 ± 0.12 vs. 1.75 ± 0.12, *α2*: 1.38 ± 0.11 vs. 1.30 ± 0.15. The MSE index decreased consistently from the non-diabetic to the type 1 diabetic group (5.31 ± 1.10 vs. 3.29 ± 0.83, *p <* 0.001); higher indices correlated with lower %CV values (*r =* -0.313, *p <* 0.001). In a subgroup of type 1 diabetes patients, insulin pump therapy significantly decreased SD1 (-0.85 mmol/l), SD2 (-1.90 mmol/l), and AFE (-16.59 mmol^2^/l^2^), concomitantly with %CV (-15.60). The MSE index declined from 3.09 ± 0.94 to 1.93 ± 0.40 (*p* = 0.001), whereas the exponents *α1* and *α2* did not. On multivariate regression analyses, SD1, SD2, SFE, and AFE emerged as dominant predictors of TIR (*β* = -0.78, -1.00, -0.29, and -0.58) but %CV as a minor one, though *α1* and MSE failed. In the regression models, including SFE, AFE, and *α2* (*β* = -0.32), %CV was not a significant predictor. Poincaré plot descriptors provide additional information to conventional variability metrics and may complement assessment of glycemia, but complexity measures produce mixed results.

## Introduction

Glucose variability (GV), as based on the amplitude of continuously recorded glycemic profiles, is an essential factor in the clinical control of diabetes, and high amplitudes in glucose excursions represent an independent predictor of hypoglycemia (Monnier et al., 2011). Moreover, GV may be a risk factor for the development of chronic diabetes complications (Nalysnyk et al., 2010). Several indices were introduced to measure GV (Rodbard, 2009), but these classical indices only consider the amplitude of the glucose signal, i.e., the global variability, and neglect any time component (Kovatchev and Cobelli, 2016). A few GV metrics, containing a time component are known. For example mean of daily differences (Molnar et al., 1972), mean absolute glucose change (Hermanides et al., 2010; Kohnert et al., 2013), and continuous overlapping net glycemic action (McDonnel et al., 2005), but they firmly correlate to the amplitude-only-based indices. A new metric, glycemic variability percentage, recently introduced by Peyser et al. (2018), gives weight to the amplitude as well as the frequency of glucose fluctuations. However, the limitation of all these indices is that they emanate from linear analyses methods and thus fail to measure the complexity or structural variability of glucose time series. The theory of non-linear dynamics provides the basis for analysis of structural variability in complex systems (Schubert, 2013). Consequently, variability analysis of physiological signals may either comprise evaluation by metrics from linear or non-linear methods. However, only non-linear analysis techniques provide access to the dynamics of regulatory systems.

Several researchers developed multiple measures of variability to assess the degree and patterns of physiological signal variation over time intervals in health and disease. Voss et al. (2009) and Bravi et al. (2011) have identified several domains of variability including geometric, information, and fractal scaling domains. We selected measures of non-linear dynamics from three different variability domains proposed by Bravi et al., 2011). These include Poincaré plots (Kovatchev et al., 2005; Crenier, 2014), multiscale entropy (Chen et al., 2014; Costa et al., 2002, 2014), and detrended fluctuation analysis (Yamamoto et al., 2010; Ogata et al., 2012; Khovanova et al., 2013; Thomas et al., 2015) for the variability analysis of glucose time series (**Table 1**). These techniques have recently found application in analyzing the dynamics of glucose time series from patients with diabetes mellitus. The results of these studies collectively showed reduced dynamics of blood glucose variations in patients with diabetes as compared with non-diabetic subjects (Ogata et al., 2012; Chen et al., 2014; Crenier, 2014; Costa et al., 2014; Khovanova et al., 2013; Kohnert et al., 2017).

**Table 1:** Techniques of variability analysis of glucose time series.

Domain	Features	Indices	Feature assumptions
Geometric	Poincaré plots features	SD1, SD2, SFE, AFE	Low dimensional representation of the dynamical attractor
Information	Multiscale entropy	MSE	The complexity changes depend on the window length used
Fractal scaling	Detrended fluctuation analysis	*α1, α2*	The SD of the detrended cumulative time series has scale-invariant properties

*Based on the classification of analysis techniques by Bravi et al. (2011)*.

Beyond traditional estimates of glycemia and glycemic variability, dynamical measures may enable assessment of several extrinsic factors and treatment modalities that can modify the intrinsic dynamics of the glucoregulatory system. However, whether such factors affect glucose dynamics or which, if any, of the dynamical measures, could complement traditional clinical measures of glycemic variability in the assessment of diabetes control is not known.

Herein, we address these problems by examining glucose dynamics in type 1 and type 2 diabetes. We compare classical measures of glycemic variability with indices from different domains of variability and investigate their interrelationships. Finally, we evaluate their contribution to the quality of glycemic control and potential clinical significance.

## Materials and Methods

### Study Design

The present study is a cross-sectional investigation that used historical data. We conducted a retrospective analysis of ambulatory continuous glucose monitoring (CGM) profiles recorded with the second-generation MiniMed CGM system (Northridge, CA, United States) set at a sampling rate of one glucose measurement every 5 min. We analyzed the CGM data using the MiniMed Solution Software (Version 2b, Medtronic MiniMed) and utilized established measures of glucose dynamics, glycemic variability, and glycemic control. Study participants had received glucose sensors placed on their abdomen. We used a minimum of four blood glucose meter calibrations per day and a mean duration of 69-h continuous monitoring. We excluded data not meeting the validity criteria of the manufacturer (≥three paired sensor/meter readings and mean absolute difference ≤ 28%).

### Study Subjects and Collection of CGM Data

The CGM data were collected for a total of 177 study participants: Twenty-two patients with type 1 diabetes (T1D), 143 with type 2 diabetes (T2D), and 12 non-diabetic control subjects (ND). All subjects participated in the Diabetiva Program (Augstein et al., 2010), an integrated national diabetes care network. The study participants entered the type and amount of food consumed into their logbooks during CGM. The entrance of consumed food enabled calculation of carbohydrate intake per day, according to standard tables containing the nutrient composition with carbohydrate exchange units (Metternich, 2008). Of the T2D patients 42 had diet alone and of those assigned to oral therapy 63 had received common oral agents only or combinations thereof. Eighteen patients had insulin plus oral antidiabetes agents, and 20 patients received insulin alone. T1D patients (*n* = 22) had been treated with multiple daily insulin injections (MDI) of short- and long-acting insulin. A subgroup of these patients (*n* = 10) switched to continuous subcutaneous insulin injections (CSII). Patients after CSII are not included in the characteristics of the total study cohort. Eighty-five percent of all patients had taken blood pressure lowering medication. Data were not included in this evaluation if patients had severe diabetes complications or decompensated glycemic control with glycated hemoglobin (HbA1c) values > 10% (86 mmol/mol).

The original study had obtained ethical approval and required no further approval for this retrospective data analysis.

### Linear Analysis

As the primary measures of GV, we computed the percentage of coefficient of variation of glucose (%CV) and standard deviation (SD) from the data obtained and averaged the data over a 48-h CGM period (Rodbard, 2011). The glucose exposure metrics included mean glucose and glycosylated hemoglobin (HbA1c). To assess the quality of glycemic control, we computed the time (h/day) in the target range (Rodbard, 2018) and defined a range of 3.9–8.9 mmol/l as acceptable for clinical practice (Bergenstal et al., 2013).

### Non-linear Analysis

Forty-eight hour CGM profiles obtained during recordings were used for calculation of dynamical parameters. We applied the standard Poincaré plot (PCP), which is a scattergram constructed by locating data points from the CGM time series on the coordinate plane according to the pairing *G_(t)_, G_(t)_+Δt*. *G_(t)_* is the glucose level at time *t*, and *Δt* is the time delay, which is a multiple of the sampling time of the signal. We probed *Δt* values of 30, 60, and 120 min but found *Δt* = 60 min most suitable to represent the PCP geometry characteristics for our study groups and used the code created by Crenier (2014) to compute the pairs of coordinates defining the PCPs. *SD1* and *SD2* statistics (Brennan et al., 2001), enabled quantification of the plots. The PCP measures included the minor axis of the fitting-ellipse (SD1) defined as the dispersion of data perpendicular to the line of identity and along the major axis (SD2) of the ellipse. Further PCP-derived metrics were the shape (SFE) and area (AFE) of the fitting ellipse calculated as *SFE = SD2/SD1* and *AFE = π^∗^SD1^∗^SD2* (Crenier, 2014). Of note, although SD1 and SD2 quantify more or less linear rather than non-linear features (Brennan et al., 2001; Schulz and Voss, 2017), we formally include these indices here in differentiating them from traditional measures of global GV.

The analysis of multiscale entropy (MSE) for the CGM sequences utilized the previously described procedure (Chen et al., 2014; Costa et al., 2014). This procedure comprised: (1) derivation of a set of time series from the original glucose signal on different time scales using the coarse-graining technique, (2) computation of sample entropy (SampEn) with standard parameter values for each coarse-grained time series. We chose the window length *m* = 2, the sensitivity criterion *r* = 0.15 times the SD, and the data length *N* = 576 within the entire coarse-grained sequence with the broadest scale factor set at *M* = 5. Thus, the length of the coarse-grained data (Humeau-Heurtier, 2015) at this scale factor contained 115 glucose samples. We calculated SampEn for the scales 1 to 5, using the mse.c program available at https://www.physionet.org/physiotools/mse/tutorial/.

The complexity index was defined as the sum of these SampEn values.

We also analyzed the CGM time series by calculating the detrended fluctuation analysis (DFA) according to the standard method, as described by (Yamamoto et al., 2010), which involves the integration of the time series and dividing it into intervals of equal size *n*. Integration of the time series was performed as follows:

$$y(k)=\sum_{t-1}^{k}\left[B(i)-B_{ave}\right]$$

*F(n)* is the calculated detrended fluctuation represented as the root-mean-square fluctuation from the trend summed up for all boxes (*B*) with *B(i)* as each point in the time series and *B_ave_* as the average of the whole series.

$$F(n)=\sqrt{\frac{1}{N}\sum_{k=1}^{N}{\left[y(k)-y_{n}(k)\right]}^{2}}$$

*n:* size of the time segments (windows) of the integrated curve
*F(n):* the measure of the difference between the integrated curve and the regression lines
*y(k)*: the value at each individual point of the integrated curve
*y_n_(k)*: the value of the regression line at the point
*N*: the total number of data points

Plots drawn with *log F(n)* on the y-axis and *log(time window)* permitted computation of the α exponents and constructing the slope of the line relating *F(n)* to *log(time window).* Because of the crossover phenomenon observed in the regression line *α* (Peng et al., 1995), we split the regression line into two regions, the short-term (*α1*) and long-term (*α2*) range. Alpha 1 represents the slope of the regression within 1.25 h calculated as *n* = 2–16 points and *α2* the slope of the regression over 1.25 h from 16 to 144 data points.

### Statistical Analysis

We categorized the patients into type 1 and type 2 diabetes and included a control group of healthy participants. We used one-way analysis of variance (ANOVA) and the *t*-test with Bonferroni–Holm correction for control of multiple pairwise comparisons. The two-tailed paired Student’s *t*-test permitted comparison between MDI and CSII data. Variables are presented as means ± SD and their statistical significance by a two-tailed test. Diabetes duration is given as median (25th – 75th) percentile. Spearman’s correlation revealed the strength of associations between dynamical indices and linear regression analyses and their associations with conventional GV measures. Multiple linear regression analysis used a core model (standardized regression coefficient denoted by *β*) that consisted of the following covariates: age, sex, diabetes duration, body mass index, carbohydrate intake, and antidiabetic therapy (CORE MODEL). Antidiabetic therapy was coded: 1 = none, 2 = diet with/without oral agents, 3 = oral agents with/without insulin and 4 = insulin alone. Stepwise forward selection identified confounding variables (McNamee, 2005). The variance inflation factor (VIF) and Durbin-Watson statistic ensured the absence of confounding effects. Results at *p* < 0.05 were statistically significant. We applied the Statistical Package for the Social Sciences software package (version 17.0; SPSS, Chicago, IL, United States) for statistical analyses.

## Results

### Characteristics of Study Subjects

The summary of demographic and clinical characteristics of the study cohort in **Table 2** shows that patients with T2D were significantly older (65.4 ± 8.2 years) than those with T1D (43.3 ± 15.2 years) or the ND control subjects (44.3 ± 12.4 years), but the age difference between the T1D and ND group was not statistically significant. Diabetes duration (years) was shorter in T2D [7.0 (3.0 – 12.0)] than in T1D [20.5 (14.8 – 29.0)], and body mass index was higher in T2D than in T1D patients (30.3 ± 4.8 vs. 25.3 ± 3.9 kg/m^2^). However, carbohydrate intake and hemoglobin A1c (HbA1c %) were lower in T2D patients than in patients with T1D; HbA1c (6.8 ± 1.0 vs. 7.7 ± 0.9), whereas mean glucose levels did not significantly differ (*p =* 0.37) between these two diabetes groups. As expected, the global GV measured by %CV was markedly higher (*p <* 0.001) in the group of T1D patients than in the T2D (36.9 ± 8.6 vs. 20.2 ± 7.4) and ND (15.7 ± 3.5) groups. Likewise, SD was significantly higher in the T1D than in the T2D and ND group. Consistent with the data on glucose exposure and GV, the time spent in target range was significantly longer (*p* < 0.001) in the ND than in the T2D and T1D group (23.4 ± 1.0 vs. 17.4 ± 6.2 vs. 13.2 ± 3.8).

**Table 2:** Demographic and metabolic characteristics of diabetic patients and control subjects.

Characteristic	Type 1 Diabetes	Type 2 Diabetes	Non-diabetes	*P*- value
Patients (*n*)	22	143	12	
Sex (male/female)	11/11	91/52	5/7	
Age (years)	43.3 ± 15.2	65.4 ± 8.2^∗∗∗,+++^	44.3 ± 12.4	<0.001
Diabetes duration (years)	20.5 (14.8 – 29.0)	7.0 (3.0 – 12.0)^∗^	NA	<0.001
Body mass index (kg/m^2^)	25.3 ± 3.9	30.3 ± 4.8^∗∗∗^	27.1 ± 4.1	<0.001
Carbohydrate intake (g/day)	211.8 ± 46.6	138.8 ± 50.7^∗∗,+++^	185.6 ± 35.3	<0.001
**Glucose exposure**		
Hemoglobin A1C (%)	7.7 ± 0.9^+++^	6.8 ± 1.0^∗∗∗,+++^	5.0 ± 0.3	< 0.001
Hemoglobin A1C (mmol/mol)	61	51	31	
Mean glucose (mmol/l)	8.0 ± 1.7^+++^	7.8 ± 2.0^+++^	5.4 ± 0.5	<0.001
**Glucose variability**		
Coefficient of variation (%)	36.9 ± 8.6^+++^	20.2 ± 7.4^∗∗∗^,^+^	15.7 ± 3.5	<0.001
Standard deviation (mmol/l)	2.9 ± 0.7^+++^	1.6 ± 0.7^∗∗∗,+++^	0.9 ± 0.2	<0.001
**Quality of glycemic control**		
Time in target range (h/day)	13.2 ± 3.8^+^	17.4 ± 6.2^∗,+^	23.4 ± 1.0	<0.001

*Data are mean ± SD or median (25th – 75th percentile) values. (NA), not applicable. The groups were compared using analysis of variance (ANOVA) and *t*-test with Bonferroni–Holm correction. Differences between the groups: ^∗∗^*p* < 0.01, ^∗∗∗^*p* < 0.001 vs. type 1 diabetes and ^+^*p* < 0.05, ^+++^*p* < 0.001vs. non-diabetes*.

### The Dynamics of CGM Tracings in the Study Groups

**FIGURE 1:**
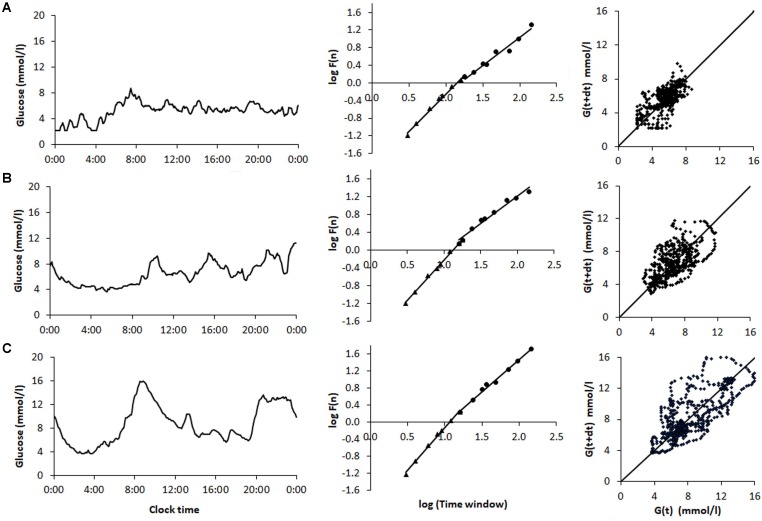
Analysis of samples of 48-h continuous glucose monitoring tracings obtained from **(A)** a non-diabetic control subject (ND), **(B)** a patient with type 2 diabetes (T2D) on sulfonylurea treatment, and **(C)** a type 1 diabetic (T1D) patient treated with multiple insulin injections. The columns (from left to right) show the CGM profiles, detrended fluctuation analysis, and Poincaré plots. The glycemic characteristics and the dynamical indices derived from the glucose time series are shown in **Table 6**. Note the increase in the Poincaré indices SD1, SD2, and AFE, the decrease in MSE as well as the increasing short- (*α1*) and long-term (*α2*) fractal scaling exponent when moving from the non-diabetic subject down to the T1D patient.

Comparison of sample CGM tracings (**Figure 1**) obtained from a non-diabetic control subject (**Figure 1A**) a patient with T2D (**Figure 1B**), and T1D patient (**Figure 1C**) exemplified that the selected dynamical indices are capable of expressing differences in glucose dynamics between individual patients. Despite well-controlled diabetes, as reflected by HbA1c < 7.0% and mean glucose values < 9.4 mmol/l, significant glycemic fluctuations in the CGM time series were evident in the two diabetic patients. There SD2, AFE, *α* exponents increased, and MSE values decreased, indicating altered glucose dynamics as compared with that of the non-diabetic sample. Note, high PCP metrics and low MSE index values correlated with large glycemic fluctuations and loss of the information content of the glucose signal.

### Dynamical Indices in Diabetic Patients and Non-diabetic Control Subjects

**FIGURE 2:**
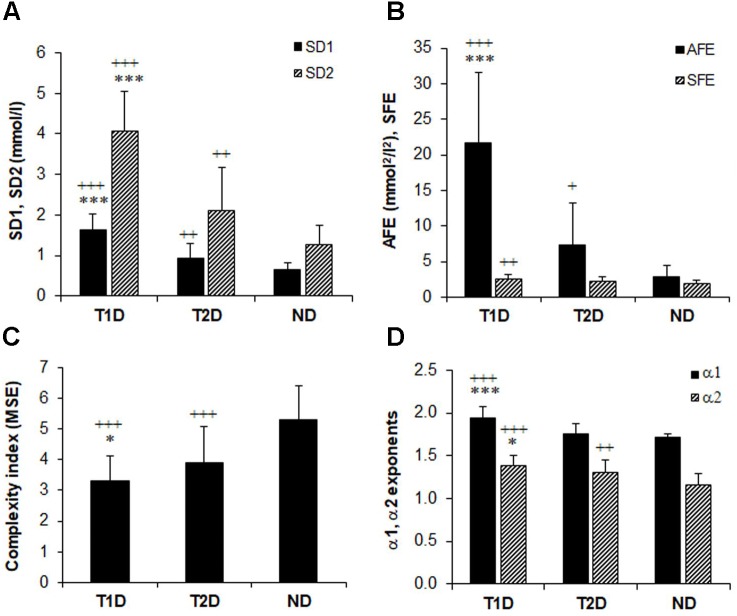
Comparison of glucose variability indices in non-diabetes (ND), type 2 (T2D), and type 1 diabetes (TD1) from the geometric, information, and fractal scaling domains. Analysis of variance (*ANOVA*) and *t*-test with Bonferroni–Holm correction gave the between-group differences as indicated: ^∗^*p* < 0.05, ^∗∗∗^*p* < 0.001 vs. type 2 diabetes and ^++^*p* < 0.01, ^+++^*p* < 0.001 vs. non-diabetes.

**Figure 2** shows that the selected non-linear GV indices in diabetic patients are significantly different (*p* < 0.05) from those in non-diabetic subjects (ND). Moreover, except for SFE, all other metrics of PCP geometry were higher in T1D than in patients with T2D (**Figures 2A,B**). The SFE index did not differ between the T1D and T2D group 2.51 ± 0.58 vs. 2.26 ± 0.64, *p* = 0.08). On the contrary, the MSE index values (**Figure 2C**) increased significantly (*p* < 0.001) between groups when moving from T1D (3.29 ± 0.83) to T2D (3.89 ± 1.19) and finally to the ND group (5.31 ± 1.10). Lower MSE indices correlated with higher %CV values (-0.313, *p* < 0.001). These changes indicate a loss of complexity in the glucose time series of diabetic patients. The two DFA *α* exponents in **Figure 2D** were higher in diabetes compared to non-diabetes, but among both diabetes groups, were higher in patients with T1D than in those with T2D: *α1* (1.95 ± 0.12 vs. 1.75 ± 0.12, *p* < 0.001) and *α2* (1.38 ± 0.11 vs. 1.30 ± 0.15, *p* = 0.017).

### Correlations Among Indices From Different Variability Domains

When we investigated the associations between the classical PCP indices by Spearman’s correlation analysis (**Table 3**), we found strong correlations of SD1 and SD2 with the AFE index (*r* = 0.776–0.805, *p* < 0.001 for all). We noticed weak negative associations between SD1 and SFE (*r* = -0.167, *p* = 0.026), whereas those with SD2 (0.604, *p* < 0.001 were stronger. The associations among MSE and the PCP indices were moderate but inverse for SD1, SD2, SFE, and AFE, (*r =* -0.432 to -0.564, *p* < 0.001), and with the exponent *α1* (*r* = -0.401, *p* < 0.001) and *α2* (*r =* -0.385, *p* < 0.001).

**Table 3:** Spearman correlation coefficients among measures of glucose dynamics.

		Poincaré plot				Multiscale	Detrended fluctuation	
						entropy	analysis	
			
		SD1	SD2	SFE	AFE	MSE	*α1*	*α2*
Poincaré plot	SD1 (short term)	1						
	SD2 (long term)	**0.872**	1					
	SFE (shape)	-0.167	**0.604**	1				
	AFE (area)	**0.776**	**0.805**	**0.365**	1			
Multiscale entropy	MSE	**-0.432**	**-0.549**	**-0.437**	**-0.564**	1		
Detrended fluctuation analysis	α1 (short term)	**0.468**	**0.454**	0.160	**0.489**	**-0.401**	1	
	α2 (long term)	0.270	**0.600**	**0.780**	**0.433**	**-0.385**	0.236	1

*Correlations at the significance level of *p* < 0.001 are given in bold face; those at *p* < 0.05 (two-tailed) in regular type face*.

### Relationships Between Dynamical Indices and Conventional Measures of Global Glucose Variability

Linear regression analysis of dynamical variables against conventional metrics of glycemic variability (**Table 4**) indicated that the PCP descriptors SD1, SD2, and AFE, with the exception of SFE, have a consistently closer and positive relationship with %CV (*β =* 0.78–0.82, *p <* 0.001) than the DFA α exponents (*β* = 0.47 and 0.41, *p* < 0.001). In contrast, the complexity index, MSE, has a weak, negative relationship (*β* = -0.36 and -0.38, *p* < 0.001) with %CV and SD. These results clearly show that numerically higher PCP metrics and DFA exponents relate to larger glucose fluctuations, whereas lower complexity index values correlate with higher glycemic variability.

**Table 4:** Linear regression analysis of indices of glucose dynamics against conventional measures of glycemic variability Coefficient of Variation (%CV) and Standard deviation (SD).

		%CV			*SD*		
		*β*	R^2^_adj_	*P-value*	*β*	R^2^_adj_	*P-value*
Poincaré plot	SD1	0.82	0.67	<0.001	0.90	0.81	<0.001
	SD2	0.82	0.67	<0.001	0.94	0.88	<0.001
	SFE	-0.34	0.11	<0.001	-0.44	0.19	<0.001
	AFE	0.78	0.60	<0.001	0.86	0.74	<0.001
Multiscale entropy	MSE	-0.36	0.13	<0.001	-0.38	0.14	<0.001
Detrended	α1	0.47	0.22	<0.001	0.43	0.14	<0.001
fluctuation analysis	α2	0.41	0.16	<0.001	0.47	0.22	< 0.001

### Dynamical Indices as Determinants of the Quality of Glucose Control

We performed multiple regression analyses to assess the independent effects of glucose dynamics on TIR as the quality measure of glycemic control. We included %CV as the conventional, linear measure of GV and the covariates age, sex diabetes duration, body mass index, carbohydrate intake, and antidiabetic therapy (**Supplementary Table 1**, see link on this article). In the fully adjusted regression models (model 2–5, 8) the dynamical indices were associated with TIR. Out of these, models 2, 3, and 5 achieved an adjusted *R^2^* of 0.39, 0.50, and 0.31 with SD1 (*β* = -0.78), SD2 (*β* = -1.00), and AFE (*β* = -0.58); %CV *β* = 0.34, 0.47, and 0.11, respectively. The statistical significance for SD1, SD2, and AFE was *p* < 0.001. These regression analyses revealed that SD1, SD2, and AFE were the most powerful predictor variables of the quality of glycemic control presented as TIR. SFE (*β =* -0.29) and *α2* (*β =* -0.32) were also significant (both *p* < 0.001) but weaker predictors. The covariates age, sex, diabetes duration, body mass index, carbohydrate intake, and antidiabetic therapy, except for carbohydrate intake in model 4 and 8, failed to contribute significantly. MSE (model 6) was not a significant predictor. In the regression models integrating the variables AFE, SFE, and *α2*, %CV failed to contribute to TIR. The variance inflation factors (≤3.5) and the Durban-Watson statistic (1.8 – 2.1) confirmed the absence of significant collinearity.

### Glucose Dynamics Before and After Switching From Multiple Daily Insulin Injections to Continuous Subcutaneous Insulin Infusions

**FIGURE 3:**
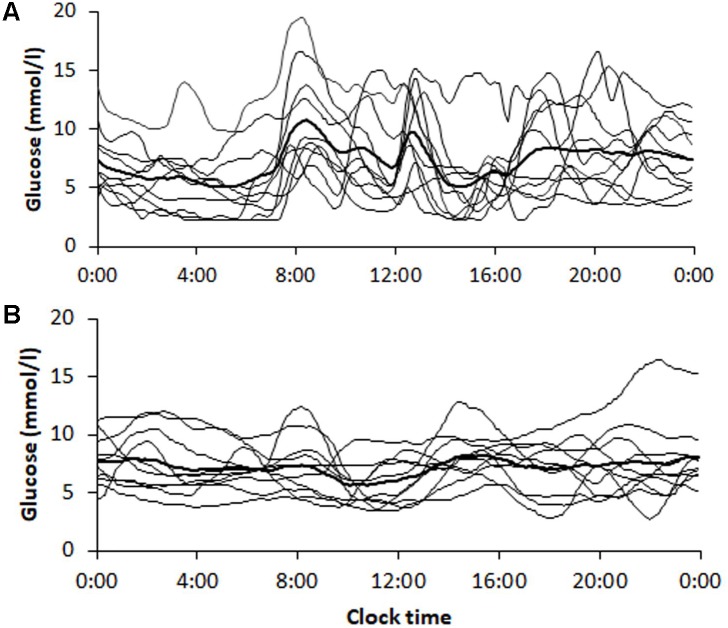
Continuous glucose monitoring tracings obtained from patients (*n* = 10) with type 1 diabetes **(A)** before and **(B)** 6 months after initiation of insulin pump therapy. Tracings are shown for each patient with the average curve in bold. The corresponding dynamic and glycemic measures are shown in **Table 5**.

The transition from multiple daily insulin injections (MDI) to continuous subcutaneous insulin infusions (CSII) in a subgroup of 10 patients with T1D, reduced SD1, SD2, and the AFE index except for SFE (**Table 5**). The significant reduction by roughly 50% in SD1, SD2, and AFE indicates an overall improvement in PCP geometry. Whereas the MSE index decreased (3.09 ± 0.94 vs. 1.93 ± 0.40, *p* = 0.001), the DFA scaling exponents *α1* (2.04 ± 0.06 vs. 2.09 ± 0.02, *p* = 0.05) and *α2* (1.43 ± 0.11 vs. 1.57 ± 0.36, *p* = 0.16) did not significantly vary. These latter results suggest a further loss of complexity and a non-significant change in fractal-like behavior of the glucose time series after initiation of insulin pump therapy. HbA1c (range 7.3 – 10.3% at baseline) and the mean glucose levels (range 6.2 – 12.1 mmol/l at baseline) did not significantly change. As **Table 5** further shows, the amplitude-based glucose fluctuations, measured as %CV and SD, declined markedly (*p* = 0.003 and 0.010, respectively). Likewise, the quality of diabetes control ameliorated, as TIR increased from 13.0 to 17.7 h/day (*p* = 0.021). This result is consistent with the CGM profiles in **Figure 3**, demonstrating lower glycemic amplitudes and better glycemic control after the commencement of CSII therapy (**Figure 3B**).

**Table 5:** Comparison of dynamic and glycemic measures in type 1 diabetic patients before and after initiation of continuous subcutaneous insulin infusion therapy.

	Before CSII	At 6 months after	*P*-value
		initiation of CSII	
**Dynamic measures**		
SD1 (mmol/l)	1.66 ± 0.37	0.81 ± 0.19	<0.001
SD2 (mmol/l)	4.37 ± 0.74	2.47 ± 0.90	<0.001
SFE	2.75 ± 0.74	3.07 ± 0.86	0.17
AFE (mmol^2^/l^2^)	23.07 ± 7.18	6.48 ± 3.32	<0.001
MSE	3.09 ± 0.94	1.93 ± 0.40	0.001
*α1*	2.04 ± 0.06	2.09 ± 0.02	0.05
*α2*	1.43 ± 0.11	1.57 ± 0.36	0.16
**Glycemic measures**		
HbA1c (%)	8.2 ± 0.85	7.7 ± 0.51	0.07
Mean glucose (mmol/l)	7.4 ± 1.2	7.5 ± 1.6	0.83
CV (%)	39.9 ± 8.5	24.3 ± 6.8	0.003
SD (mmol/l)	2.9 ± 0.6	1.8 ± 0.7	0.010
Time in range (h/day)	13.0 ± 3.0	17.7 ± 5.3	0.021

*The number of patients was *n* = 10. Data are mean ± SD values. *P*-values are two-tailed*.

**Table 6:** Summary of characteristics and metrics of the continuous glucose monitoring profiles shown in **Figure 1** for a non-diabetic control subject (ND), a patient with type 2 diabetes (T2D), and a type 1 diabetic patient (T1D).

ID	Group	Diabetes duration	HbA1c (%)	Mean glucose	CV (%)	SD1	SD2	AFE	MSE	α1	α2
		(years)		(mmol/l)		(mmol/l)	(mmol/l)	(mmol^2^/l^2^)			
777773	ND	NA	5.1	5.6	23.42	0.81	2.00	5.08	5.47	1.81	1.32
128701	T2D	13	6.1	6.1	26.27	1.11	2.39	8.31	4.66	1.83	1.24
125264	T1D	29	6.8	8.7	32.37	1.48	3.67	17.06	2.86	2.02	1.53

## Discussion

Glucose time series may differ in individual diabetic patients despite comparable HbA1c and mean glucose levels because such clinical, linear measures are not appropriate to reveal the inherent dynamics of the glucoregulatory system.

We demonstrate that several indices derived from the geometric, information, and fractal scaling domains of variability techniques can characterize the variability of glucose time series in health and diabetes. Previous studies in the literature (Yamamoto et al., 2010; Khovanova et al., 2013; Chen et al., 2014; Costa et al., 2014; Weissman and Binah, 2014), using various non-linear signal processing techniques, reported that glucose dynamics appears reduced in patients with diabetes compared with non-diabetic subjects. However, it is not known whether the type and severity of diabetes or factors such as age, diabetes duration, BMI, and carbohydrate intake or antihyperglycemic therapy may affect the dynamical behavior of glucose time series. Regarding the natural history of diabetes, immune-mediated destruction of the pancreatic β-cells leading to an irreversible loss of the β-cell mass characterizes T1D, whereas in T2D a progressive decline of β-cell function over time occurs with rising insulin resistance and deterioration of glucose regulation. Because of the diverse pathogenic mechanisms, T1D needs insulin and is difficult to control, but those patients with T2D are able to manage their disease mostly with a variety of oral antihyperglycemic agents.

The indices derived from PCP and DFA analysis in the present study provided qualitatively similar results with respect to the differentiation of the glucose time series dynamics between the two types of diabetes, i.e., the values for these indices be significantly lower in the T2D than in the T1D group but were lowest in the ND group. In contrast, the complexity index calculated from MSE is highest in the ND group and lowest in the T1D group. These data are also compatible with the increase in glucose fluctuations (reduced non-linear autocorrelation) and thus with the diminished glycemic stability observed in the glucose profile structure of our T1D and T2D patient samples (**Figure 1**). In so far PCP descriptors in T1D and healthy control subjects are concerned, this is in agreement with a report by Crenier (2014). Regarding the ratio of long-term to short-term glucose time series variability, SFE was correspondingly higher in patients with T1D than in the non-diabetic subjects. Numerically high PCP indices unequivocally point toward dynamical instability in the glucoregulation. Nevertheless, the indices SD1, SD2, and AFE of PCP analysis quantify linear rather than non-linear features of the underlying time series (Brennan et al., 2001; Fishman et al., 2012). Consistent with the changes that occurred in the PCP geometry, the decreased MSE, and the altered DFA plots with increased *α1* and α2 exponents observed in the T1D group further indicate significant alterations in the feedback mechanism that is less able to diminish glucose fluctuations in patients with T1D than in those with T2D.

Our previous results demonstrated that the β-cell function is an independent predictor of glucose time series dynamics as measured by the DFA alpha exponents (Kohnert et al., 2014). Thus, the reduced glucose dynamics in the T1D versus the T2D patient group allows the assumption that worsening of the glucoregulation is partly due to the loss of the β-cell secretory capacity, whereas the remaining β-cell reserve prevents such derailment in T2D. The variability indices from the different domains correlated weakly or moderately to one another. As one could expect, the strongest correlations existed between the PCP indices within the geometric domain. The unexpectedly weak correlation across the variability domains suggests that the indices are not interchangeable. These correlations are in a way comparable with those found for the cardiac interbeat time series (Bassi et al., 2015) because the information retrieved from PCP and from DFA analysis show structural correlations of the underlying dynamics. By the multivariate regression analyses, we disclosed that the measures of glucose profile dynamics are independent predictors of the quality of glucose control, as defined by the time spent in target range (TIR). The analyses showed that the PCP indices SD1, SD2, and AFE along with %CV were independent determinants of TIR (**Supplementary Table 1**). Sex, age, diabetes duration, BMI, carbohydrate intake, and antidiabetic treatment had, if any, no substantial influence. Of note, we found that SD1, SD2, and AFE explained 35, 44, and 29%, respectively, of the interindividual variance in TIR compared to an additional 3–8% defined by the %CV. The variables SFE and *α2*, even significant in the regression models, were weaker predictors, explaining 17 and 21% of TIR. Moreover, the regression models (4, 5, and 8) including SFE, AFE, and *α2* demonstrate that these measures predicted the quality of glycemic control, whereas the overall glycemic variability as measured by %CV was not a significant predictor. The MSE did not determine TIR which indicates that this index is not useful in the assessment of the quality of glycemic control. Therefore, the evaluated indices do not reflect purely the global GV, although they relate to conventional measures of GV. Methods analyzing the fluctuation of glucose time series are not detecting the same phenomena as those methods that identify amplitude-based glycemic variability. Indeed, a loss in glucose time series dynamics gives rise to increased overall glycemic variability (Garcia Maset et al., 2016). Although strongly correlated with SD (in our study *r* = 0.876, *p* < 0.0001), we chose %CV for our regression models as the standard metric of GV in clinical research (Rodbard, 2018) to compare its effect on TIR with those indices from the different variability domains. We used TIR as an established and clinically useful indicator of the quality of glycemic control, reflecting the time in predefined target ranges (Bergenstal et al., 2013 and Rodbard, 2018).

Finally, we investigated the dynamics of glucose time series in a cohort of T1D patients in response to insulin pump therapy. CSII yielded a marked improvement in the PCP geometry–consistent with the report by Crenier (2014), except for the SFE descriptor, with a corresponding decrease in glycemic variability (calculated as %CV and SD) and increased quality of glycemic control as evaluated using TIR. Although HbA1c did not significantly change and mean glucose not markedly drop (Vogt et al., 2016), one may conclude that overall glycemia improved because of reduced glycemic variability and increased TIR. Of note, however, the MSE index decreased, whereas the DFA long-term exponent *α2* tended to increase. This is an unexpected result, and we do not have any plausible explanation, because healthier glycemic dynamics are associated both with higher MSE values and lower α exponents (see **Figures 2C,D**). Nevertheless, these finding suggests that even 6 months of CSII could not halt the loss of glucose time series complexity and fractal structure in glucose dynamics. In other words, CSII therapy is inappropriate to reverse glucose dynamics to those of non-diabetic subjects. We assume that owing to the absolute β-cell insulin deficiency in T1D, the glucoregulatory system remains insufficient to correct defective glucose dynamics. Islet transplantation rather than insulin pump therapy would offer restoration of the β-cell function (Vantyghem et al., 2014). Whether such therapy can restore glucose complexity and the fractal structure is not known and requires appropriate clinical studies.

This study has limitations in as much as it is retrospective in nature, and the number of subjects in the T1D and ND group is relatively small. Furthermore, the follow-up time in the patients after insulin pump therapy appears too short to allow restitution of glucose complexity and the fractal structure of glucose time series. In patients with T2D, for example, β-cell function began to increase not until after 12 months of CSII therapy (Choi et al., 2013). The strength of the investigation is the use of indices from different variability domains and classical GV measures as well as the inclusion of both patients with T1D and T2D to enable comparison of glucose dynamics between distinct types of diabetes.

In summary, this study provides evidence that glucose time series dynamics differ between the two primary forms of diabetes. The loss of complexity is more pronounced in T1D than in T2D, which we anticipate is due to differences in the β-cell pathology. Insulin pump therapy for 6 months can not reverse multiscale dynamics toward those of non-diabetic subjects because of the failure to mimic healthy patterns of insulinemia. Our findings, which corroborate and extend previous work by others, also emphasize the need for using an ensemble of indices from various variability domains to characterize glucose time series more specifically. Moreover, we show that a combination of several dynamical metrics and classical GV measures has the potential to assess both the natural glucoregulatory system and quality of blood glucose control which may help in approaching diabetes treatment on a personalized basis.

## Author Contributions

K-DK contributed to the study conception, data analysis, interpretation, statistical analyses, and wrote the manuscript, added to the revision of the manuscript for intellectual content and approval. PH contributed to the data collection, review, interpretation, revision of the manuscript for intellectual content, and support of the paper. LV and PA devoted to the study conception, design, and approval of the document. ES is the guarantor of this work and, as such, took responsibility for the integrity of data and the accuracy of the data analysis.

## Conflict of Interest Statement

The authors declare that the research was conducted in the absence of any commercial or financial relationships that could be construed as a potential conflict of interest.

## References

[B1] Augstein P. Vogt L. Kohnert K. D. Heinke P. Salzsieder E. (2010). Translation of personalized decision support into routine diabetes care. *J. Diabetes Sci. Technol.* 4 1532–1539. 10.1177/19322681000400631 21129352 PMC3005067

[B2] Bassi D. Arakelian V. M. Goncalves Mendes R. Rossi Caruso F. C. Bonjorno Junior J. C. Lopes Zangrando K. T. (2015). Poor glycemic control impacts linear and non-linear dynamics of heart rate in dm type 2. *Rev. Bras. Med. Esporte* 21 313–317. 10.1590/1517-869220152104150003

[B3] Bergenstal R. M. Ahmann A. J. Bailey T. Beck R. W. Bissen J. Buckingham B. (2013). Recommendations for standardizing glucose reporting and analysis to optimize clinical decision making in diabetes: the ambulatory glucose profile. *Diabetes Technol. Ther.* 15 198–211. 10.1089/dia.2013.0051 23448694

[B4] Bravi A. Longtin A. Seely J. E. (2011). Review and classification of variability analysis techniques with clinical applications. *Biomed. Eng. Online* 10:90 10.1186/1475-925X-10-90 PMC3224455 21985357

[B5] Brennan M. Palaniswami M. Kamen P. (2001). Do existing measures of Poincaré plot geometry reflect nonlinear features of heart rate variability? *IEEE Trans. Biomed. Eng.* 48 1342–1347. 10.1109/10.959330 11686633

[B6] Chen J. L. Chen P. F. Wang H. M. (2014). Decreased complexity of glucose dynamics in diabetes: evidence from multiscale entropy analysis of continuous glucose monitoring system data. *Am. J. Physiol. Regul. Integr. Comp. Physiol.* 307 R179–R183. 10.1152/ajpregu.00108.2014 24808497

[B7] Choi S.-B. Lee J.-H. Lee J.-H. Kim S. Han S.-D. Kim I.-H. (2013). Improvement of ß-cell function after achievement of optimal glycaemic control via long-term continuous subcutaneous insulin infusion therapy in non-newly diagnosed type 2 diabetic patients with suboptimal glycaemic control. *Diabetes Metab. Res. Rev.* 29 473–482. 10.1002/dmrr.2416 23592489

[B8] Costa M. Goldberger A. L. Peng C. K. (2002). ). Multiscale entropy analysis of complex physiologic time series. *Phys. Rev. Lett.* 89:068102 10.1103/PhysRevLett.89068102 12190613

[B9] Costa M. D. Henriques T. Munshi M. N. Segal A. R. Goldberger A. L. (2014). Dynamical glucometry: use of multiscale entropy analysis in diabetes. *Chaos* 24:033139 10.1063/1.4894537 PMC5848691 25273219

[B10] Crenier L. (2014). Poincaré plot quantification for assessing glucose variability from continuous glucose monitoring systems and a new risk marker for hypoglycemia: application to type 1 diabetes patients switching to continuous subcutaneous insulin infusion. *Diabetes Technol. Ther.* 16 247–254. 10.1089/dia.2013.0241 24237387

[B11] Fishman M. Jacono F. J. Park S. Jamasebi R. Thungtong A. Loparo K. A. (2012). A method for analyzing temporal patterns of variabilityof a time series from Poincaré plots. *J. Appl. Physiol.* 113 297–306. 10.1152/japplphysiol.01377.2010 22556398 PMC3404703

[B12] Garcia Maset L. Blasco Gonzales L. Llop Furquet G. Montes Suay F. Hernandes Marco R. (2016). Study of glycemic variability through time series analyses (detrended fluctuation analysis and Poincaré plot) in children and adolescents with type 1 diabetes. *Diabetes Technol. Ther.* 18 719–724. 10.1089/dia.2016.0208 27728773

[B13] Hermanides J. Vriesendorp T. M. Bosman R. J. Zandstra D. F. Hoekstra J. B. DeVries J. H. (2010). Glucose variability is associated with intensive care unit mortality. *Crit. Care Med.* 38 838–842. 10.1097/CCM.0b013e3181cc4be9 20035218

[B14] Humeau-Heurtier A. (2015). The multiscale entropy algorithm and its variants: a review. *Entropy* 17 3110–3123. 10.3390/e17053110

[B15] Khovanova N. A. Khovanov I. A. Shabno L. Griffiths F. Holt T. A. (2013). Characterisation of linear predictability and non- stationarity of subcutaneous glucose profiles. *Comput. Methods Programs Biomed.* 110 260267 10.1016/j.cmpb.2012.11.009 23253451

[B16] Kohnert K. D. Heinke P. Fritzsche G. Vogt L. Augstein P. Salzsieder E. (2013). Evaluation of the mean absolute glucose change as a measure of glycemic variability using continuous glucose monitoring. *Diabetes Technol. Ther.* 6 448–454. 10.1089/dia.2012.0303 23550553

[B17] Kohnert K. D. Heinke P. Vogt L. Augstein P. Salzsieder E. (2014). Decling ß-cell function is associated with the lack of long-range negative correlations in glucose dynamics and increased glycemic variability: a retrospective analysis in patients with type 2 diabetes. *J. Clin. Transl. Endocrinol.* 1 192–199. 10.1016/j.jcte.2014.09.003 29159101 PMC5685022

[B18] Kohnert K. D. Heinke P. Vogt L. Augstein P. Thomas A. Salzsieder E. (2017). Association of blood glucose dynamics with antihyperglycemic treatment and glycemic variability in type 1 and type 2 diabetes. *J. Endocrinol. Invest.* 40 1201–1207. 10.007/s40618-017-0682-2 28484994

[B19] Kovatchev B. Cobelli C. (2016). Glucose variability: timing, risk analysis, and relationship to hypoglycemia in diabetes. *Diabetes Care* 39 502–510. 10.2337/dc16-0841 27208366 PMC4806774

[B20] Kovatchev B. P. Clarke W. L. Breton M. Brayman K. McCall A. (2005). Quantifying temporal glucose variability in diabetes via continuous glucose monitoring: mathematical methods and clinical application. *Diabetes Technol. Ther.* 7 849–862. 10.1089/dia.2005.7.849 16386091

[B21] McDonnel C. M. Donath S. M. Vidmar S. I. Werther G. A. Cameron F. J. (2005). A novel approach to continuous glucose analysis utilizing glycemic variation. *Diabetes Technol. Ther.* 7 253–263. 10.1089/dia.2005.7.253 15857227

[B22] McNamee R. (2005). Regression modelling and other methods to control confounding. *Occup. Environ. Med.* 62 500–506. 10.1136/oem.2002.001115 15961628 PMC1741049

[B23] Metternich K. (2008). *Die Diabetes-Journal-Naehrwert-Tabelle: BE, KE und Kalorien auf einen Blick*. Mainz: Verlag Kirchheim+CoGmbH.

[B24] Molnar G. D. Taylor W. F. Ho M. M. (1972). Day-to-day variation of continuously monitored glycaemia: a further measure of diabetes instability. *Diabetologia* 8 342–348. 10.1007/BF01218495 4641791

[B25] Monnier L. Wojtuscizyn A. Colette C. Owens D. (2011). The contribution of glucose variability to asymptomatic hypoglycemia in persons with type 2 diabetes. *Diabetes Technol. Ther.* 13 813–818. 10.1089/dia.2011.0049 21561372

[B26] Nalysnyk L. Hernandez-Medina M. Krishnarajah G. (2010). Glycaemic variability and complications in patients with diabetes mellitus: evidence from a systematic review of the literature. *Diabetes Obes. Metab.* 12 288–298. 10.1111/j.1463-1326.2009.01160.x 20380649

[B27] Ogata H. Tokuyama K. Nagasaka S. Tsuchita T. Kusaka I. Ishibashi S. (2012). The lack of long-range negative correlations in glucose dynamics is associated with worse glucose control in patients with diabetes mellitus. *Metabolism* 61 1041–1050. 10.1016/j.metabol.2011.12.007 22304838

[B28] Peng C. K. Havlin S. Stanley H. E. Goldberger A. L. (1995). Quantification of scaling exponents and crossover phenomena in nonstationary heartbeat time series. *Chaos* 5 82–87. 10.1063/1.166141 11538314

[B29] Peyser T. A. Balo A. K. Buckingham B. A. Hirsch I. B. Garcia A. (2018). Glycemic variability percentage: a novel method for assessing glycemic variability from continuous glucose monitor data. *Diabetes Technol. Ther.* 20 6–16. 10.1089/dia.2017.0187 29227755 PMC5846572

[B30] Rodbard D. (2009). Interpretation of continuous glucose monitoring data: glycemic variability and quality of glycemic control. *Diabetes Technol. Ther.* 11(Suppl. 1), S55–S67. 10.1089/dia.2008.0132 19469679

[B31] Rodbard D. (2011). Glycemic variability: measurement and utility in clinical medicine and research-one viewpoint. *Diabetes Technol. Ther.* 11 1077–1080. 10.1089/dia.2011.0104 21815751

[B32] Rodbard D. (2018). Metrics to evaluate quality of glycemic control: comparison of time in target range, hypoglycemic, and hyperglycemic ranges with “risk indices”. *Diabetes Technol. Ther.* 20 325–334. 10.1089/dia.2018.0092 29792750

[B33] Schubert P. (2013). The application of nonlinear methods to characterize human variability from time series. *Ger. J. Sports Med.* 64 132–140. 10.5960/dzsm.2012.064

[B34] Schulz S. Voss A. (2017). “Symbolic dynamics, Poincaré plot analysis and compression entropy estimate complexity in biological time series,” in *Complexity and Nonlinearity in Cardiovascular Signals*, ed. Valenza G. (Berlin: Springer International Publishing), 45–85.

[B35] Thomas F. Signal M. Chase J. G. (2015). Using contiuous glucose monitoring data and detrended fluctuation analysis to determine patient condition: a review. *J. Diabetes Sci. Technol.* 9 1327–1335. 10.1177/1932296815592410 26134835 PMC4667316

[B36] Vantyghem M. C. Raverdy V. Belavoine A. S. Defrance F. Caiazzo R. Arnalsteen L. (2014). Continuous glucose monitoring after islet transplantation in type 1 diabetes: an excellent graft function (ß-score greater than 7) is required to abrogate hyperglycemia, whereas a minimal function is necessary to supress severe hypoglycemia (ß-score greater than 3). *J. Clin. Endocrinol. Metab.* 97 E3–E6. 10.1210/jc.2012-2115 PMC3485599 22996144

[B37] Vogt L. Kohnert K. D. Heinke P. Thomas A. Salzsieder E. (2016). Use of the KADIS-CSII PROGRAM for adjusting insulin pump therapy in type 1 diabetes. Bulletin of the Karaganda University. *Biol. Med. Geogr. Ser.* 4 25–33.

[B38] Voss A. Schulz S. Schroeder R. Baumert M. Caminal P. (2009). Methods derived from nonlinear dynamics for analysing heart rate variability. *Phil. Trans. R. Soc. A* 367 277–296. 10.1098/rsta.2008.0232 18977726

[B39] Weissman A. Binah O. (2014). The fractal nature of blood glucose fluctuations. *J. Diabetes Complications* 28 646–651. 10.1016/j.jdiacomp.2014.05.009 24996977

[B40] Yamamoto N. Kubo Y. Ishizawa K. Kim G. Moriya T. Yamanouchi T. (2010). Detrended fluctuation analysis is considered to be useful as a new indicator for short-term glucose complexity. *Diabetes Technol. Ther.* 12 775–783. 10.1089/dia.2010.0059 20809679

