# Supplementary material for: Applications of Variability Analysis Techniques for Continuous Glucose Monitoring Derived Time Series in Diabetic Patients
Source: Front Physiol. 2018 Sep 6;9:1257. doi: 10.3389/fphys.2018.01257 (PMC6136234; doi:10.3389/fphys.2018.01257)
Supplement: Supplementary file 1 [file Table_1.DOC]

***Supplementary Material:***

**Applications of Variability Analysis Techniques for Continuous Glucose Monitoring Derived Time Series in Diabetic Patients**

*Klaus-Dieter Kohnert^1*^, Peter Heinke^1^, Lutz Vogt^2^, Petra Augstein^1,3^, and Eckhard Salzsieder^1^*

*^1^ Institute of Diabetes "Gerhardt Katsch", Karlsburg, Germany*

*^2^ Diabetes Service Center, Karlsburg, Germany*

*^3^Heart and Diabetes Medical Center, Karlsburg, Germany*

*Correspondence:

Klaus-Dieter Kohnert

kohnert@diabetes-karlsburg.de

**Table 1**│Multiple linear regression analyses for variability metrics predicting quality of

glycemic control as presented by the Time in target range

|  | ***ß*** | ***p-*value** | **R²_adjusted_** |
| --- | --- | --- | --- |
| **CORE MODEL** |  |  | 0.18 |
| Age (years) | -0.07 | 0.43 |  |
| Sex (male/female) | 0.01 | 0.87 |  |
| Diabetes duration (years) | -0.09 | 0.34 |  |
| Body mass index (kg/m²) | 0.11 | 0.12 |  |
| Carbohydrate intake (g/day) | -0.23 | 0.004 |  |
| Antidiabetic treatment | -0.29 | 0.003 |  |
| **MODEL 1: CORE MODEL + CV%** |  |  | 0.20 |
| Age (years) | -0.09 | 0.28 |  |
| Sex (male/female) | 0.02 | 0.82 |  |
| Diabetes duration (years) | 0.08 | 0.38 |  |
| Body mass index (kg/m²) | -0.14 | 0.044 |  |
| Carbohydrate intake (g/day) | -0.20 | 0.013 |  |
| Antidiabetic treatment | -0.18 | 0.10 |  |
| CV(%) | -0.23 | 0.015 |  |
| **MODEL 2: CORE MODEL + CV% + SD1** |  |  | 0.39 |
| Age (years) | -0.06 | 0.41 |  |
| Sex (male/female) | 0.02 | 0.80 |  |
| Diabetes duration (years) | -0.05 | 0.49 |  |
| Body mass index (kg/m²) | -0.13 | 0.042 |  |
| Carbohydrate intake (g/day) | -0.08 | 0.30 |  |
| Antidiabetic treatment | -0.13 | 0.17 |  |
| CV (%) | 0.34 | 0.003 |  |
| SD1(mmol/l) | -0.78 | < 0.001 |  |
| **MODEL 3: CORE MODEL + CV% + SD2** |  |  | 0.50 |
| Age (years) | -0.04 | 0.54 |  |
| **Table 1**│continued |  |  |  |
|  | ***ß*** | ***p-*value** | **R²_adjusted_** |
| Sex (male/female | 0.02 | 0.78 |  |
| Diabetes duration (years) | -0.01 | 0.93 |  |
| Body mass index (kg/m²) | -0.07 | 0.25 |  |
| Carbohydrate intake (g/day) | -0.11 | 0.10 |  |
| Antidiabetic treatment | -0.03 | 0.74 |  |
| CV (%) | 0.47 | < 0.001 |  |
| SD2 (mmol/l) | -1.00 | < 0.001 |  |
| **MODEL 4: CORE MODEL + CV% + SFE** |  |  | 0.27 |
| Age (years) | -0.08 | 0.29 |  |
| Sex (male/female) | 0.02 | 0.81 |  |
| Diabetes duration (years) | -0.07 | 0.44 |  |
| Body mass index (kg/m²) | -0.10 | 0.16 |  |
| Carbohydrate intake (g/day) | -0.23 | 0.003 |  |
| Antidiabetic treatment | -0.12 | 0.22 |  |
| CV (%) | -0.14 | 0.13 |  |
| SFE | -0.29 | < 0.001 |  |
| **MODEL 5: CORE MODEL + CV% + AFE** |  |  | 0.31 |
| Age (years) | -0.04 | 0.57 |  |
| Sex (male/female) | 0.02 | 0.71 |  |
| Diabetes duration (years) | -0.01 | 0.87 |  |
| Body mass index (kg/m²) | -0.18 | 0.006 |  |
| Carbohydrate intake (g/day) | -0.10 | 0.19 |  |
| Antidiabetic treatment | -0.08 | 0.44 |  |
| CV (%) | 0.11 | 0.29 |  |
| AFE (mmol²/l²) | -0.58 | < 0.001 |  |
| **MODEL 6: CORE MODEL + CV% + MSE** |  |  | 0.20 |
| Age (years) | -0.08 | 0.33 |  |
| Sex (male/female) | 0.02 | 0.77 |  |
| Diabetes duration (years) | -0.07 | 0.42 |  |
| Body mass index (kg/m²) | -0.14 | 0.06 |  |
| Carbohydrate intake (g/day) | -0.20 | 0.012 |  |
| Antidiabetic treatment | -0.15 | 0.15 |  |
| CV (%) | -0.20 | 0.032 |  |
| MSE | 0.09 | 0.23 |  |
| **MODEL 7: CORE MODEL + CV% + α1** |  |  | 0.20 |
| Age (years) | -0.09 | 0.31 |  |
| Sex (male/female) | 0.01 | 0.83 |  |
| Diabetes duration (years) | -0.08 | 0.37 |  |
| Body mass index (kg/m²) | -0.14 | 0.05 |  |
| Carbohydrate intake (g/day) | -0.20 | 0.013 |  |
| Antidiabetic treatment | -0.18 | 0.10 |  |
| CV (%) | -0.23 | 0.016 |  |
| α1 | 0.01 | 0.90 |  |
| **MODEL 8: CORE MODEL + CV% + α2** |  |  | 0.27 |
|  |  |  |  |
| **Table 1**│continued |  |  |  |
|  | ***ß*** | ***p-*value** | **R²_adjusted_** |
| Age (years) | -0.09 | 0.23 |  |
| Sex (male/female) | 0.02 | 0.77 |  |
| Diabetes duration (years) | -0.06 | 0.52 |  |
| Body mass index (kg/m²) | -0.05 | 0.46 |  |
| Carbohydrate intake (g/day) | -0.24 | 0.003 |  |
| Antidiabetic treatment | -0.09 | 0.36 |  |
| CV (%) | -0.11 | 0.22 |  |
| α2 | -0.32 | < 0.001 |  |

*ß denotes the standardized regression coefficient*
